# Effectiveness of telepsychiatry in college students: A cross sectional evaluation

**DOI:** 10.6026/973206300214304

**Published:** 2025-12-15

**Authors:** Murali M.R, Mallikarjun Samala, Rahul Tiwari, Heena Dixit Tiwari, Prashant M.C, Tohid Ali, Afroz Kalmee Syed

**Affiliations:** 1 Department of Psychiatry, JSS Academy of Higher Education & Research, Mysore, Karnataka, India; 2 Piedmont Rockdale Hospital & Family, First Primary Clinic, Atlanta, GA, USA; 3 Department of Oral and Maxillofacial Surgery, RKDF Dental College and Research Centre, Sarvepalli Radhakrishnan University, Bhopal, Madhya Pradesh, India; 4 Programme Officer, Blood Cell, Commissionerate of Health and Family Welfare, Government of Telangana, Hyderabad, India; 5 Department of Oral Medicine and radiology, RKDF Dental College and Research Centre, Sarvepalli Radhakrishnan University, Bhopal, Madhya Pradesh, India; 6 Department of Oral and Maxillofacial Pathology, Writing and Publications, Tenali andhra Pradesh, India

**Keywords:** Telepsychiatry, college students, effectiveness, satisfaction, engagement

## Abstract

Telepsychiatry has rapidly expanded as a response to the growing mental health needs of college students. This cross-sectional evaluation surveyed 376 students across three universities regarding usage, satisfaction and effectiveness of telepsychiatry. Results showed significant reductions in depression and anxiety symptoms, high satisfaction and reduced missed appointments, particularly with video-based consultations. Privacy, connectivity and rapport were key barriers. Thus, we show that telepsychiatry is an effective and acceptable alternative to in-person care, though implementation should address engagement and privacy concerns.

## Background:

Mental health problems among college students have increased substantially over the past decade, with depression and anxiety among the most prevalent concerns [1]. Access to timely psychiatric support remains challenging due to stigma, limited campus resources and long waiting times [2]. Telepsychiatry - delivering psychiatric assessment and treatment via secure video or phone platforms-offers a solution to these barriers by improving access, reducing travel and offering flexibility [3]. Although evidence supports telepsychiatry in adult and general populations [4, 5] studies specifically targeting college students are fewer. Existing findings suggest comparable symptom improvement and high satisfaction, though challenges around privacy, digital access and engagement persist [6, 7]. The COVID-19 pandemic catalysed widespread adoption of telehealth in universities, providing a natural context for evaluation [8]. Therefore, it is of interest to describe the effectiveness and acceptability of telepsychiatry among college students within this evolving care landscape.

## Methods:

## Study design and participants:

We conducted a cross-sectional survey between March-June 2024 across three universities in India. Eligible participants were undergraduate or postgraduate students aged 18-25 years who had at least one telepsychiatry consultation in the prior 6 months.

## Sampling and recruitment:

Participants were recruited via university counseling centers and email invitations. A total of 412 students responded; after excluding incomplete responses, 376 were included in the final analysis.

## Data collection:

A structured questionnaire was developed, piloted and administered online.

It included:

[1] Demographics: age, gender, academic year.

[2] Telepsychiatry usage: number of sessions, modality (video/phone).

[3] Effectiveness: change in symptoms measured by PHQ-9 (for depression) and GAD-7 (for anxiety).

[4] Satisfaction: Likert-scale questions (1-5).

[5] Barriers: privacy, internet connectivity, rapport, stigma.

## Data analysis:

Descriptive statistics were used to summarize data. Associations between demographics and satisfaction were tested using chi-square. Independent t-tests compared PHQ-9/GAD-7 changes between video and phone users. Analyses were performed using SPSS v27.

## Results:

Of the 376 participants, mean age was 20.8 ± 2.1 years; 58% were female. Most (72%) attended video-based sessions, while 28% used phone consultations. Table 1 (see PDF) demonstrates the demographic and utilization profile of students engaging in telepsychiatry. The sample was predominantly female, with a mean age of 20.8 years, reflecting the typical undergraduate demographic. Most participants used video consultations rather than phone calls, suggesting a preference for visual interaction. Over 60% had more than three sessions, indicating sustained engagement with telepsychiatry services. These findings underscore that video-based platforms are widely adopted among young adults, with substantial adherence to repeated sessions, supporting feasibility in university contexts. Table 2 (see PDF) highlights the effectiveness and perceived quality of telepsychiatry. Statistically significant improvements were observed in both depression (PHQ-9) and anxiety (GAD-7) scores, confirming therapeutic benefits. Satisfaction levels were high, with nearly three-quarters rating sessions as helpful, particularly among video users. Missed or canceled appointments were low, reflecting better continuity compared to pre-pandemic in-person care. Despite positive outcomes, students reported challenges including privacy concerns, internet connectivity issues and weaker rapport, which emphasize the importance of structural and technological support to optimize effectiveness.

## Discussion:

This cross-sectional evaluation demonstrates that telepsychiatry is effective and well-accepted among college students, with symptom improvements comparable to existing literature [9, 10- 11]. High satisfaction rates and reduced missed appointments align with findings from large outpatient cohorts [12]. Importantly, video-based consultations yielded better outcomes than phone-based care, suggesting video should remain the preferred modality [13]. Barriers identified-notably privacy, internet reliability and rapport-mirror prior qualitative work among young adults [14, 15]. Interventions such as provision of private booths on campus, stable connectivity and clinician training to foster digital rapport may address these concerns.

## Strengths and limitations:

Strengths include a multi-university sample and standardized symptom scales. Limitations include reliance on self-report, lack of control group and potential response bias. Longitudinal studies are warranted.

## Conclusion:

Telepsychiatry is an effective, accessible and acceptable mode of care for college students, leading to significant symptom improvement, high satisfaction and reduced missed visits. Thus, universities should prioritize video-based telepsychiatry, integrate hybrid models and invest in privacy and engagement supports to optimize outcomes.
